# Application of the PDCA cycle in companion diagnostic PD-L1 SP263 immunohistochemical testing

**DOI:** 10.1186/s12885-025-14572-4

**Published:** 2025-07-12

**Authors:** Xiaoying Liu, Feicheng Yang, Kun Wang, Zhe Zhang, Penghui Dai, Jie Zhao, Zhihong Chen

**Affiliations:** Department of Pathology, Hunan Provincial People’s Hospital and The First Affiliated Hospital of Hunan Normal University, Changsha, 410005 China

**Keywords:** PDCA cycle, Immunohistochemical testing, Quality control, Immunotherapy

## Abstract

The aim of this study was to investigate application of the PDCA (Plan-Do-Check-Action) cycle in companion diagnostic PD-L1 SP263 immunohistochemical (IHC) testing. By using scientific quality control methods, the accuracy and consistency of the experimental results were ensured.

**Methods** A total of 279 cases of PD-L1 SP263 IHC testing conducted in our hospital from May 2021 to December 2021 were collected. The PDCA cycle method was applied, and quality control data before and after its implementation were analyzed and compared.

**Results** The application of the PDCA cycle effectively strengthened quality control management that significantly improved the accuracy and consistency of PD-L1 SP263 IHC testing results.

**Conclusion** PD-L1 SP263 testing has value as both a complementary and companion diagnostic test for immunotherapy in malignant tumors, with the accuracy of its results being particularly crucial. The testing process involves pre-analytical, analytical, and post-analytical phases. The application of the PDCA cycle provides a robust scientific approach to aid quality control management throughout the PD-L1 SP263 testing process.

## Introduction

Programmed death-ligand 1 (PD-L1) plays a crucial role in the immune invasion of malignant tumors and has become an important biomarker for immunotherapy in patients with malignancies [1–3]. PD-L1 is expressed highly in various types of malignant tumors, such as lymphoma, malignant melanoma, lung cancer, urothelial carcinoma, hepatocellular carcinoma, and breast cancer [4]. The FDA has approved PD-L1 as a complementary diagnostic factor for certain specific indications of PD-L1 inhibitors and also as a companion diagnostic for other indications [5]. Current clinical data show that monoclonal antibodies such as Atezolizumab, Pembrolizumab, Avelumab, and Durvalumab, which have been approved by the FDA, are effective in the immunotherapy of locally advanced and metastatic urothelial carcinoma [6]. Atezolizumab, approved by the FDA, is also effective for adjuvant treatment in patients with stage II-IIIA non-small cell lung cancer (NSCLC) who have undergone surgical resection and platinum-based chemotherapy, with PD-L1 SP263 expression detected in ≥ 1% of tumor cells [7]. In addition, the clone SP263 has been approved by the FDA as a companion diagnostic reagent for this indication [8].

The detection of PD-L1 expression levels in malignant tumors is gaining increasing attention, especially in the era of precision medicine. The results of PD-L1 immunohistochemical (IHC) testing can directly guide clinical decisions by selecting patients who may benefit from immunotherapy. For pathology laboratories, PD-L1 IHC testing is an issue that warrants emphasis and attention [9]. The IHC testing process involves multiple steps, with many factors being able to influence the results, including tissue preparation, operational methods, and interpretation of the result. In daily clinical practice, challenges exist in the implementation, standardization, and reproducibility of these tests. Establishing a standardized quality control system is therefore crucial for PD-L1 IHC testing. In this regard, the application of the PDCA (Plan-Do-Check-Act) cycle can provide a more scientific and comprehensive quality control management approach for the entire PD-L1 IHC testing process. This approach can significantly improve the accuracy and consistency of PD-L1 IHC test results and provide reliable information for immunotherapy in various malignancies.

In this context, the current study collected 279 cases that underwent PD-L1 SP263 IHC testing, with the aim of investigating the value and impact of applying the PDCA cycle. The results of the study will guide the establishment of standard operating procedures (SOPs) for quality control in PD-L1 SP263 IHC testing in laboratories, ultimately leading to improved management of scientific quality control.

## Materials and methods

### Materials

A series of 279 cases who underwent PD-L1 SP263 IHC testing using the VENTANA immunohistochemistry instrument combined with OptiView DAB at our hospital from May 2021 to December 2021 were collected. Among these cases, 103 from May to July 2021 served as the control group (before application), while 176 from August to December 2021 constituted the experimental group (after application). The aim of this retrospective study was to analyze and summarize quality control issues encountered in PD-L1 SP263 IHC testing and to develop more comprehensive quality control guidelines. The study was approved by the Ethics Committee of Hunan Provincial People’s Hospital.

### Methods

#### Plan

A quality control management team was established to ensure the accuracy and consistency of the PD-L1 SP263 IHC testing results. First, the testing process and SOPs were defined. The scoring scheme for PD-L1 SP263 IHC staining was adopted based on the recommendations provided by the manufacturer of the reagent [10]. In addition, the department established strict SOPs for the pre-analytical, analytical, and post-analytical steps. As a critical biomarker in tumor immunotherapy, the accurate detection of PD-L1 SP263 is essential for clinicians to be able to formulate treatment plans. Compared to traditional IHC testing techniques, PD-L1 SP263 testing requires higher technical standards. Prior to the application of the PDCA cycle, the rate for excellent PD-L1 SP263 IHC testing was only 82%. After implementation, the target rate for excellent PD-L1 SP263 testing was set at 98-100%.

#### Do (i.e., implementation of the PDCA cycle)

From May to July 2021, 103 cases were tested as the control group. The issues identified in PD-L1 SP263 testing included false-negative staining (including positive control tissue and placenta) caused by instrument malfunctions and operator errors, and weak staining and non-specific staining that were influenced by multiple factors, primarily the numerous steps involved in immunohistochemistry, including fixation and slide preparation, which introduced various interfering factors leading to abnormal staining [11]. To address these problems encountered in the control group, the PDCA cycle was applied to scientifically manage quality control for the 176 cases tested from August to December 2021. This enabled timely analysis and resolution of the issues.

**Fig. 1:**
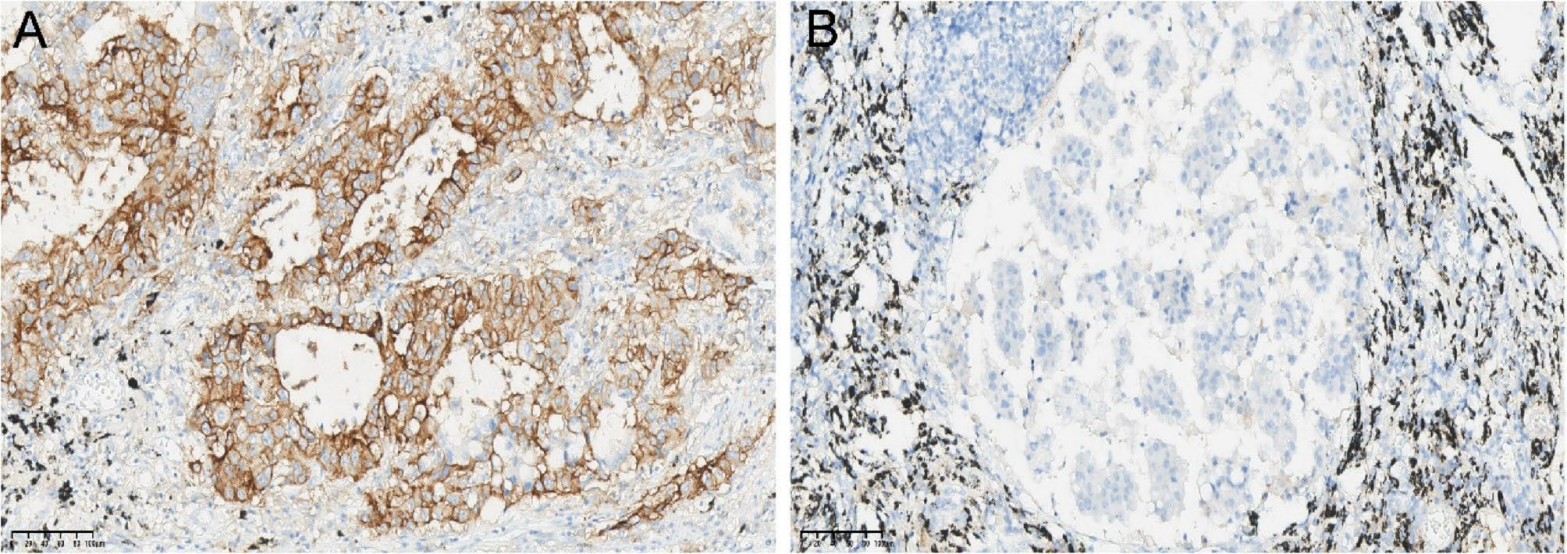
High expression of PD-L1 SP263 immunohistochemical staining in the primary lesion of lung adenocarcinoma (**A**) Negative expression of PD-L1 SP263 immunohistochemical staining in the metastatic lesion of the same case of lung adenocarcinoma (**B**)

##### Improvements for avoiding weak staining

It was recommended to perform PD-L1 testing on paraffin-embedded tumor tissues or metastatic carcinoma sections. Due to tumor heterogeneity, the status of PD-L1 expression may differ between primary and metastatic sites (Figs. 1A and B). PD-L1 testing was not recommended for cytology or decalcified tissues. Proper fixation was crucial and included the use of 3.7% buffered neutral formalin as the fixative, with a fixation time of 6–48 h. Insufficient or excessive fixation could lead to weak staining or false-negative results. The thickness of the sections was set at 3–4 μm as thicker sections may cause artificially enhanced staining. Staining also needed to be performed promptly after sectioning. The sections should not have been stored at room temperature for more than a few weeks or alternatively stored in a 4 °C refrigerator, as prolonged storage may lead to protein degradation and antigen loss, resulting in weak staining or false negative results.

##### Improvements for tissue detachment

High-quality slides that were more compatible with the immunohistochemistry instrument were used in the test, while supervision of the consultation slides was strengthened. If the quality of consultation slides was considered to be suboptimal, the slides were optimized before use by soaking them in detergent for 10 min and then in 15% milk powder for 10 min. This enhanced hydrophilicity and reduced the likelihood of detachment or false negative results. Proper baking of slides was essential, with the slides baked at 65 °C for 30 min or 60 °C overnight. For fragile, brittle, hard or poorly dehydrated tissues, the time for baking was extended appropriately, but not exceeding 24 h at 60℃, in order to prevent detachment and staining failure.

##### Improvements for non-specific staining

First, interfering colored substances in the tissue itself were excluded. When selecting the tissue blocks, tissues that were degenerated, necrotic, contained minimal tumor tissue, or were poorly fixed were excluded. The sections need to be complete, as thin as possible, approximately 3–4 μm, and free of folds, knife marks, or bubbles. Non-specific staining can be caused by excessive sensitivity of the reagent kit, over-antigen retrieval, failure to block endogenous peroxidase, prolonged antibody incubation, excessive DAB development, or insufficient hematoxylin counterstaining.

##### Improvements for human factors

Strict use of approved instrument-matched reagent kits was mandated to ensure that the reagents were used within their validity period. Laboratory personnel strictly followed the storage instructions provided in the reagent manuals. The operators checked the reagent bottles daily before use to verify reagent volume, ensure smooth operation of the bottle springs, and check for crystallization at the bottle openings. Regular training and assessments were conducted for immunohistochemistry laboratory personnel to ensure consistency among operators that emphasized teamwork and strict adherence to laboratory protocols. Only those personnel who passed the assessments were allowed to perform the experimental operations.

##### Improvements for instrument malfunctions

The manufacturer was required to perform regular maintenance and servicing of the immunohistochemistry instrument. The operators conducted basic checks on the instrument daily before use to ensure correct functioning and to avoid staining failures caused by malfunctions.

#### Check of operating procedures

After application of the PDCA cycle and during the operation of the PD-L1 SP263 IHC test, all staff were encouraged to conduct self-checks and mutual checks of the operating procedures. Any issues identified were reported promptly to the quality control management team and then recorded. The person in charge of the immunohistochemistry laboratory performed strict quality control and documentation of all the experimental results to evaluate the stability and accuracy of the experiments. This included checking the staining of the tissue itself and negative and positive controls. The final interpretation was made by a pathologist, with the department director overseeing the entire process. Through multiple applications of the PDCA cycle, potential issues were identified and corrected in a timely manner. This continuously improved the skills and quality of the laboratory staff. After repeated PDCA cycles, a SOP for PD-L1 SP263 IHC testing was established and followed strictly to ensure the accuracy and consistency of the experimental results.

#### Actions regarding test improvement

A detailed analysis of the stage-specific issues in PD-L1 SP263 IHC testing was conducted to identify solutions and further optimize the operating procedures. This optimization aimed to prevent various factors from interfering with the experimental results and to continuously improve the quality of the testing outcomes. However, the application of the PDCA cycle to PD-L1 SP263 IHC testing was just the beginning. In the future, we plan to extend the PDCA cycle to the quality management of all IHC tests in the laboratory, ultimately covering all pathological operating procedures within the department.

### Observation indicators

The factors affecting the PD-L1 SP263 IHC testing results included weak staining, non-specific staining, tissue detachment, human factors, and instrument malfunctions. After applying the PDCA cycle, each issue was analyzed and addressed by targeted improvements.

### Statistical analysis

The statistical analyses were performed using SPSS 25.0 software. The comparison of excellent PD-L1 SP263 rates before and after the application of the PDCA cycle was performed using the chi-square test (Fisher’s exact probability test). A *P*-value < 0.05 was considered statistically significant.

## Results

**Fig. 2:**
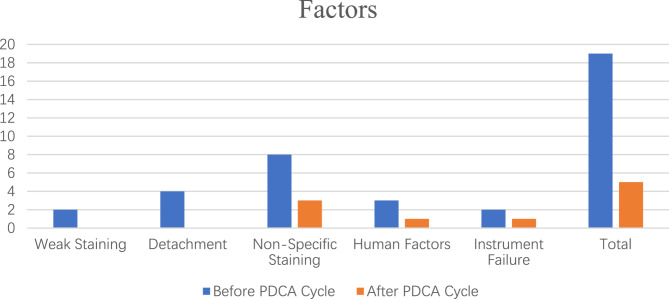
Impact of Factors on PD-L1 Expression Before and After PDCA Cycle

After multiple applications of the PDCA cycle in PD-L1 SP263 IHC testing, quality improved significantly (Table 1) and the overall rate of excellent testing increased from 82% before the cycle to 98% after the cycle (Table 2). Following implementation of multiple PDCA cycles the factors leading to IHC testing failures were reduced significantly (Fig. 2). In November and December, the rate of excellent PD-L1 SP263 testing reached 100%. Following the optimization and improvement of the process, a quality control system for PD-L1 SP263 IHC testing was established. As shown in Table 3, the SOPs for each step of the IHC testing process were adhered to rigorously, and then extended to all the IHC tests in the laboratory. As a result, the internal quality control of IHC testing was enhanced significantly.

**Table 1:** Comparison of PD-L1 SP263 immunohistochemical testing quality before and after PDCA cycle implementation

PD-L1 SP263	Total Cases	Excellent (+)*	Non-Excellent (-)*	*P*-value
**Before PDCA Cycle**	103	84	19	< 0.001
**After PDCA Cycle**	176	171	5	

Excellent (+): Cases meeting quality standards; Non-Excellent (-): Cases failing to meet quality standards. The *P*-value (< 0.001) indicates a statistically significant improvement in quality after PDCA cycle implementation

## Discussion

IHC testing is used primarily in the diagnosis of tumors, with multiple IHC tests providing significant prognostic and valuable predictive information for selected human tumors [12]. IHC testing also plays a crucial role in tumor classification, diagnosis, differential diagnosis, prognosis assessment, and immunotherapy. PD-L1 is a cell membrane protein present in tumor cells, immune system cells within the tumor microenvironment, and various healthy tissues and has a significant inhibitory role in the immune system [13]– [14]. Over the past decade, immunotherapy has become an effective cancer treatment, applicable to cancer types responsive to FDA-approved PD-1/PD-L1 antibody products [15]. Currently, PD-L1-targeted immunotherapy has been approved in many countries for treating various human cancers. However, how to select the beneficiary population for PD-L1 inhibitor treatment and to predict the therapeutic efficacy of PD-L1 inhibitors are new challenges in the era of immunotherapy. IHC detection of the PD-L1 SP263 biomarker can serve as a complementary or companion diagnostic test, providing valuable information for immunotherapy [16]. Detecting PD-L1 levels is crucial, especially in the era of precision cancer treatment [17]. The detection results can directly guide the clinical selection of appropriate immunotherapeutic drugs, while accurate detection results provide important auxiliary information for diagnostic pathologists, enabling them to issue more accurate and meaningful pathological diagnostic reports. These procedures are now widely available and are used commonly in pathology laboratories. However, the correct application of IHC procedures and interpretation of the results requires the establishment and implementation of proper quality control and quality assurance measures [18]. Scientific quality control management is therefore key in PD-L1 SP263 IHC testing, with accurate detection results relying on standardized experimental operations. However, PD-L1 SP263 IHC testing is complex and influenced by various factors, including tissue fixation, experimental steps, human factors, and machine factors. Without standardized quality control criteria, ideal results cannot be achieved, and therefore the establishment of standardized testing procedures is urgently required. For many laboratories, calibrating and validating IHC tests to achieve optimal performance remains a daunting task [19]. Therefore, establishing standardized and reproducible quality control management for IHC testing is crucial [20].

**Fig. 3:**
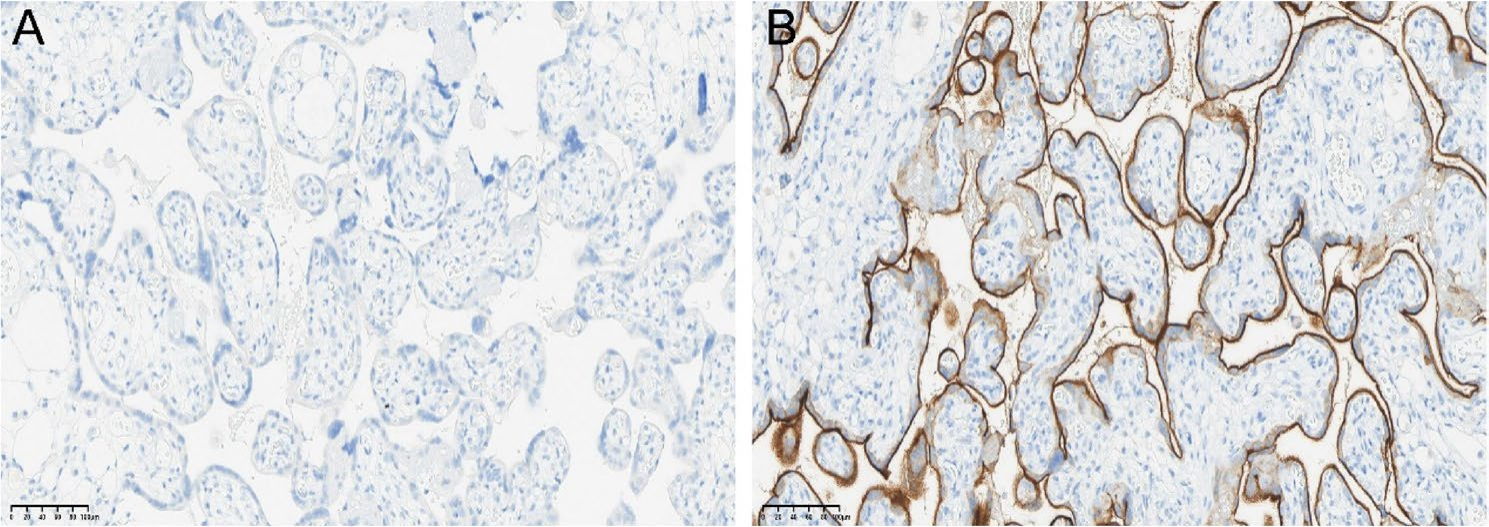
Negative control of PD-L1 SP263 immunohistochemical staining in placental tissue (**A**). Positive control of PD-L1 SP263 immunohistochemical staining in placental tissue (**B**)

The PDCA (Plan-Do-Check-Act) cycle was first proposed by Dr. Deming in 1950 [21]. The cycle includes four stages, planning, execution, checking, and action to operational management, and has been proven to be effective in various fields. Validation results confirmed that the PDCA cycle provided a scientific management framework for PD-L1 SP263 IHC testing in our laboratory as it eliminated various factors affecting detection and effectively strengthened the supervision of the entire PD-L1 SP263 IHC testing process, thereby significantly improving work efficiency and quality. Through the continuous implementation of the PDCA cycle, more scientific quality control methods have been developed. The application of the PDCA cycle in PD-L1 SP263 testing therefore enhanced quality control management across all processes and improved the quality control system and the procedure used to validate PD-L1 SP263 IHC. In addition, the cycle optimized workflows, standardized manual operations, and wherever possible, automating processes, resulted in operational errors caused by human factors being avoided. Through continuous quality monitoring and improvement, the accuracy and reproducibility of PD-L1 SP263 IHC testing can be enhanced significantly. Furthermore, accurate PD-L1 SP263 IHC test results rely on the establishment of negative and positive controls. The negative controls use rabbit monoclonal negative control immunoglobulin reagents (Fig. 3A), while positive controls use human placental tissue. Both negative and positive controls should be stained on the same slide as the target tissue. In normal staining, the trophoblast cells of the placenta show uniform moderate to strong cell membrane staining and any intensity of cytoplasmic staining, while the placental villous stroma and vasculature are negative (Fig. 3B).

The application of the PDCA cycle has ultimately standardized the operational procedures for PD-L1 SP263 IHC testing in laboratories and has continuously advanced quality control management and further improved the accuracy and consistency of the test results. The cycle has also provided reliable experimental support for personalized immunotherapy of malignant tumors, and therefore has significant clinical importance.

**Table 2:** Quality control analysis of PD-L1 SP263 immunohistochemical testing before and after PDCA cycle implementation (Cases)

Time	Total slides	Weak staining	Tissue detachment	Non-specific staining	Human factors	Instrument failure	Excellent rate
2021.5	42	1	2	3	1	1	81%
2021.6	32	0	1	3	1	1	81%
2021.7	29	1	1	2	1	0	83%
2021.8	31	0	0	1	1	1	90%
2021.9	34	0	0	1	0	0	97%
2021.10	30	0	0	1	0	0	97%
2021.11	38	0	0	0	0	0	100%
2021.12	43	0	0	0	0	0	100%

The period from May to July 2021 represents the time before application of the PDCA cycle, while the period from August to December 2021 represents the time after application of the PDCA cycle

**Table 3:** Standardized operating procedure for PD-L1 SP263 immunohistochemistry testing using the PDCA cycle

PDCA phase	Key steps	Standardized operational content.	Quality control points
**Plan**	Develop SOP	Define testing process (pre-, mid-, and post-analysis stages). Follow reagent manufacturer-recommended scoring scheme (e.g., placental tissue positive control).	SOP must cover specimen type, fixation conditions, scoring criteria; target excellence rate ≥ 98%
	Team and goal setting	Establish a QC management team, and set the excellence rate target (98-100%).	Regularly review target achievement.
**Do**	Specimen pre-treatment	Specimen type: Paraffin-embedded tumor tissue (avoid cytology/decalcified tissue). Fixation: 3.7% neutral formalin, 6–48 h Sectioning: 3–4 μm, bake at 65 °C for 30 min or 60 °C overnight.	Check fixation time, section integrity; avoid antigen degradation.
	Testing process	Use VENTANA instrument-matched reagent kit (OptiView DAB).	Daily check reagent expiry, instrument status; avoid non-specific staining.
	Personnel & instrument management	Regular operator training and assessment. Routine instrument maintenance (manufacturer + daily self-check).	Maintain maintenance logs; unqualified personnel prohibited from the operation.
**Check**	Internal QC	Each batch was tested with negative control (rabbit IgG) and positive control (placental tissue). Results of pathologist reviews.	Placental tissue must show: Moderate-strong positivity in trophoblast cell membranes. Negativity in stroma and blood vessels.
	Issue recording and feedback	Operators cross-check and record issues (e.g., weak staining, detachment. Report to QC team.	Establish issue tracking sheet; analyze root causes.
**Action**	Continuous improvement	Optimize processes based on issues (e.g., extend baking time for fragile tissue).	Update SOP and train all staff.
	Expanded application	Extend PDCA cycle to other IHC tests.	Regularly assess overall laboratory QC performance.

## Data Availability

No datasets were generated or analysed during the current study.

## Abbreviations

PDCA: Plan-Do-Check-Action
PD-L1: Programmed Death-Ligand 1
NSCLC: Non-small cell lung cancer
FDA: Food and Drug Administration
IHC: Immunohistochemical
SOP: Standard operating procedure
QC: Quality control
